# Atomic Structure of Luminescent Centers in High-Efficiency Ce-doped *w*-AlN Single Crystal

**DOI:** 10.1038/srep03778

**Published:** 2014-01-21

**Authors:** Ryo Ishikawa, Andrew R. Lupini, Fumiyasu Oba, Scott D. Findlay, Naoya Shibata, Takashi Taniguchi, Kenji Watanabe, Hiroyuki Hayashi, Toshifumi Sakai, Isao Tanaka, Yuichi Ikuhara, Stephen J. Pennycook

**Affiliations:** 1 Materials Science and Technology Division, Oak Ridge National Laboratory, Oak Ridge, TN 37831, USA; 2 Department of Materials Science and Engineering, Kyoto University, Sakyo, Kyoto 606–8501, Japan; 3 Materials Research Center for Element Strategy, Tokyo Institute of Technology, Yokohama 226–8503, Japan; 4 School of Physics, Monash University, Victoria 3800, Australia; 5 Institute of Engineering Innovation, University of Tokyo, Bunkyo, Tokyo 113–8656, Japan; 6 Japan Science and Technology Agency, PRESTO, Kawaguchi, Saitama 332–0012, Japan; 7 Advanced Key Technologies Division, National Institute for Materials Science, Tsukuba, Ibaraki 305–0044, Japan; 8 Nanostructures Research Laboratory, Japan Fine Ceramics Center, Atsuta, Nagoya 456–8587, Japan

## Abstract

Rare-earth doped wurtzite-type aluminum nitride (*w*-AlN) has great potential for high-efficiency electroluminescent applications over a wide wavelength range. However, because of their large atomic size, it has been difficult to stably dope individual rare-earth atoms into the *w*-AlN host lattice. Here we use a reactive flux method under high pressure and high temperature to obtain cerium (Ce) doped *w*-AlN single crystals with pink-colored luminescence. In order to elucidate the atomic structure of the luminescent centers, we directly observe individual Ce dopants in *w*-AlN using annular dark-field scanning transmission electron microscopy. We find that Ce is incorporated as single, isolated atoms inside the *w*-AlN lattice occupying Al substitutional sites. This new synthesis method represents a new alternative strategy for doping size-mismatched functional atoms into wide band-gap materials.

Wide band-gap materials such as diamond or III-nitride semiconductors (GaN, AlN and their alloys) are attractive for applications such as ultraviolet light-emitting diodes, lasers and high-power photonic devices^1^,^2^,^3^,^4^,^5^. For shorter wavelength optoelectronic applications, rare-earth doped III-nitrides also have significant potential for high-efficiency luminescent applications including photoemitters, phosphors and scintillators over wide wavelength ranges^6^,^7^. Wurtzite-type AlN (*w*-AlN) has one of the widest direct band-gaps of the nitrides (6.2 eV), a high thermal conductivity (~300 W m^−1^ K^−1^) and excellent physical and chemical stability^8^,^9^, and could thereby enable new electroluminescent properties in the ultraviolet or visible-light wavelength ranges. However, the key issues that must be overcome for *w*-AlN to realize its full potential are precise impurity control and effective, stable doping of luminous elements into the host lattices. Up to now, sublimation growth^10^ or hydride vapor phase epitaxy methods^11^ have been used to produce *w*-AlN single crystals. Although these methods are practically useful to synthesize millimeter size single crystals, it is difficult to control the carbon and oxygen impurity levels. Moreover, it is difficult to dope rare-earth elements into the host lattice due to the large size mismatch. As an alternative, therefore, ion implantation has been extensively used for the rare-earth doping of thin films^12^,^13^,^14^,^15^. However the resultant defect structure involving the dopants is uncontrollable and meta-stable defect structures may be introduced that are detrimental to luminescent efficiency.

Here we report the stable doping of single isolated Ce atoms into *w*-AlN single crystals. To overcome the above issues, we use a method involving a reactive flux, a temperature gradient at high pressure and temperature^16^,^17^,^18^, which results in high-quality *w*-AlN:Ce single crystals with visible-light luminescence (see Figure 1(a)). To directly identify the atomic sites of individual single Ce dopants within *w*-AlN single crystals, we use atomic-resolution scanning transmission electron microscopy (STEM) in the annular dark-field (ADF) imaging mode, which has a strong dependence on atomic number (Z-contrast)^19^ and is a powerful method to visualize single heavy dopants^18^. Combining with systematic first-principles calculations, we uncover the atomic structure of high-efficiency Ce centers in the wide band-gap *w*-AlN.

The present *w*-AlN:Ce single crystals were synthesized by the temperature gradient method at 6.5 GPa and 1600°C. The resulting sub-millimeter *w*-AlN:Ce single crystals (~0.2 mm cube) show optical transparency and the band edge luminescent feature of *w*-AlN is observed at 210 nm in cathodoluminescence (CL) spectroscopy, suggesting that the impurity level is sufficiently suppressed. Figure 1(a) shows a photoluminescence (PL) image of *w*-AlN:Ce single crystals. The homogeneous pink-colored luminescence shows that the Ce dopants are uniformly doped throughout the single crystals. A single broadband peak, observed at 600 nm in the PL spectrum of Figure 1 (b), is assigned to the 4*f* – 5*d* electron transition of Ce^3+^ dopants in a bulk material. The valence state of Ce dopants in the *w*-AlN single crystals was evaluated by Ce *M*_4,5_ X-ray absorption near edge structure (XANES). Two specific peaks in Figure 1(c) are assigned to the Ce *M*_5_ (879 eV) and *M*_4_ (897 eV) edges. By comparing to the standard XANES spectra of Ce^3+^ in CeF_3_ and Ce^4+^ in CeO_2_, the valence state of the Ce dopants in *w*-AlN is determined to be 3+.

Figure 2(a) shows a typical atomic-resolution ADF STEM image of *w*-AlN:Ce viewed along the 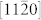 direction. The scattering strength of nitrogen is relatively weak, but the atomic dumbbell consisting of Al and N atoms is clearly resolved (the projected spacing is 1.1 Å), as is evident in Figure 2(b). Thus, we can directly determine the atomic site of the Ce dopants from the images, whether they are on Al, N or interstitial sites. As marked by the arrows in Figure 2(a), the atomic columns containing Ce dopants are observed as slightly brighter spots, and one can see that the Ce dopants are dispersed as single atoms. This isolation of single Ce dopants is critical for the optical luminescence because dopant-clusters lose radiative recombination efficiency, in particular for large clusters, owing to their local metallic properties. The present rare-earth doping process in *w*-AlN is thus highly effective, and all Ce dopants should contribute equally to the pink-colored luminescence. Note that, even after the extensive observations, we could not observe any rare-earth dimer- or trimer-clusters, as previously proposed^12^,^14^, suggesting that these clusters may be meta-stable defect structures. In the high-magnification atomic-resolution ADF STEM image of Figure 2(b), the single Ce dopant evidently substitutes for the Al site without apparent atomic displacements. This bright feature at the Al site is quantitatively reproduced in the simulated image (Figure 2(c)), where a single Ce atom has been substituted for an Al atomic site.

In order to theoretically estimate the stable Ce atom configurations in the *w*-AlN lattice, we performed systematic first-principles calculations using the Heyd-Scuseria-Ernzerhof (HSE06) hybrid functional^20^,^21^, which has been applied to Ce dopants in *c*-BN^18^ as well as a variety of solids including rare-earth compounds^22^,^23^,^24^. The formation energy (*E_f_*) of a defect is written as
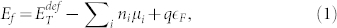
where 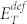 denotes the total energy of the supercell (192 atoms) with a defect in charge state *q*, *n_i_* is the number of the constituent atoms of *i*-type, and *μ_i_* and 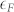 are the atomic chemical potential and the Fermi level. The chemical potentials for the *w*-AlN:Ce single crystals are not clear under the present synthesis conditions, and therefore we treated the chemical potentials as variables. *μ*_Al_, *μ*_Ce_, and *μ*_N_ are considered to range between the following extreme conditions: the Al-rich limit (*μ*_Al_ = *μ*_Al(bulk)_, *μ*_N_ = 1/2*μ*_N2_ + Δ*E_f_* (AlN), and *μ*_Ce_ = *μ*_Ce(bulk)_) and the N-rich limit (*μ*_N_ = 1/2*μ*_N2_, *μ*_Al_ = *μ*_Al(bulk)_ + Δ*E_f_* (AlN), and *μ*_Ce_ = *μ*_Ce(bulk)_ + Δ*E_f_* (CeN)), where *μ*_Al(bulk)_, *μ*_Ce(bulk)_, and *μ*_N2_ are the chemical potentials of the Al and Ce crystals and a N_2_ molecule given by the calculated total energies, and Δ*E_f_* (AlN) and Δ*E_f_* (CeN) are the calculated formation energies of *w*-AlN and CeN, −3.14 and −2.97 eV, respectively. In this calculation, we consider several defect structures and complexes: (1) Ce substitutions on the Al and N sites, Ce_Al_ and Ce_N_; (2) Ce as an interstitial at the octahedral site, Ce_i_; (3) Ce coupled with an Al-vacancy, Ce_Al_-V*^i^*_Al_ (*i* = *a*, *b*, or *c*, where Ce_Al_ and V_Al_ are located on the same *c*-plane or V_Al_ is located on the *c*-plane below or above Ce_Al_; and (4) Ce coupled with a N-vacancy, Ce_Al_-V*^j^*_N_ (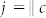 or 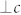, where Ce_Al_ and V_N_ are located along the direction parallel to or nearly perpendicular to the *c* axis). In addition, isolated Al and N vacancies (V_Al_ and V_N_) were considered as the dominant native defects in *w*-AlN.

Figures 3(a) and (b) show the formation energies of several defect structures and complexes considered here as a function of the Fermi level. The formation energies of V_N_ and V_Al_ (dashed lines in Figures 3(a) and (b)) are negative in the lower Fermi levels (< 1.2 eV) at the Al-rich limit and in the higher Fermi levels (> 4.4 eV) at the N-rich limit, respectively. The Fermi level cannot be located in these ranges because of carrier compensation associated with the spontaneous formation of negatively charged V_Al_ and positively charged V_N_. The XANES measurement suggests the presence of Ce^3+^ in the *w*-AlN:Ce single crystals. From the formation energy and one-electron structure analyses, the energetically stable defect structures that involve Ce^3+^ are (1) neutral Ce_Al_, 
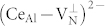
 or (Ce_Al_-V*^i^*_Al_)^3−^ at the Al-rich limit and (2) neutral Ce_Al_ or (Ce_Al_-V*^i^*_Al_)^3−^ at the N-rich limit, depending on the Fermi level. The (Ce_Al_-V*^i^*_Al_)^3−^ defect complexes are the most energetically preferable structures at a high Fermi level, but after structural relaxation the Ce atoms show large atomic shifts away from the Al sites (0.61 Å toward V_Al_, where *i* = *c* is the most stable defect structure in (Ce_Al_-V*^i^*_Al_)), which is inconsistent with the ADF STEM observations. The 
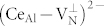
defect complex also shows a lower formation energy than Ce_Al_, but the condition is limited to very high Fermi levels (> 5.2 eV) and only found at the Al-rich limit. In the relaxed 
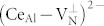
 defect structure, the Ce atom exhibits a substantial atomic shift toward the N vacancy site from the Al site (0.23 Å). On the other hand, neutral Ce_Al_ is the most energetically stable defect structure under the remaining wide range of conditions (the chemical potential only slightly affects the formation energy). As seen in the relaxed structure (Figures 3(c) and (d)), the Ce atom is located at the Al site without apparent atomic displacement, which is in good agreement with the observed ADF STEM images. Thus, we conclude that the atomic structure luminescent center in *w*-AlN is neutral Ce_Al_.

It is noted that the Al-N bond length (1.89 Å) is ~29% shorter than that of Ce-N (2.44 Å) in the compound CeN. Hence, it may appear to be impossible to accommodate an isolated Ce atom into the *w*-AlN lattice. However, in the neutral Ce_Al_ defect structure, the tetrahedrally coordinated N atoms shift their positions and the resultant Ce-N bond length (2.22 Å) is ~17% longer than that of Al-N. We note here that the theoretical formation energy of a neutral Ce_Al_ defect is comparatively high (~3 eV) and so the doping of Ce atoms into the *w*-AlN lattice would seem to be very unlikely under ambient conditions. However, the present high pressure and high temperature extreme conditions are able to overcome the high formation energy of these Ce_Al_ luminescent centers.

In summary, we have synthesized Ce-doped *w*-AlN single crystals with high efficiency pink-colored luminescent centers through an optimized high pressure, high temperature flux method. The atomic structure of the luminescent center in *w*-AlN has been determined through direct observation of atomic-resolution ADF STEM imaging combined with systematic first-principles calculations. Our findings indicate that the present synthesis method enables the stable doping of isolated single Ce atoms and could be extended to control the emission color by choosing appropriate dopant elements. Moreover, it opens a new alternative strategy for doping size-mismatched functional atoms into wide band-gap materials, overcoming the high formation energies.

## Methods

### Single crystal synthesis

The single crystals of *w*-AlN:Ce were synthesized by the temperature gradient crystal growth method at 6.5 GPa and 1600°C for 20 hours, using a modified belt-type high-pressure apparatus with a bore diameter of 30 mm (Ref. 16, 17). The source of AlN powder (Toyo Soda, Type F) placed at upper part in the sample chamber was dissolved into the molten solvent and *w*-AlN crystals were precipitated at the cooler bottom in the chamber via a spontaneous nucleation process. We used a mixture of Li_3_AlN_2_ and Ba_3_Al_2_N_4_ as a solvent (typically, 1:1 molar ratio). In order to suppress carbon and oxygen contamination, we prepared solvents at 900°C under dry nitrogen atmosphere, and then high-purity CeF_3_ powder (Rare metallic Co. Ltd, 4N grade, 0.5 wt%) as a dopant source was also mixed under the same atmosphere. The fundamental procedure for the crystal growth is similar to that of cubic boron nitride (*c*-BN) single crystals, where the solvents are Li_3_BN_2_ and Ba_3_B_2_N_4_ (Ref. 16, 17). The yields of *w*-AlN crystals are essentially 100% because the source of AlN powder was entirely dissolved into the solvents and precipitated as recrystallized *w*-AlN during growth duration, though the size of the crystals depend upon the position in the Mo sample chamber. Typical weights of the source and the solvent are 0.15 g and 0.18 g.

### Electron microscope experiments and simulations

The surface of the obtained single crystals was cleaned by hot aqua regia. To directly image Ce dopants in the *w*-AlN lattice, an important prerequisite is to prepare clean and electron-transparent thin TEM specimens (<10 nm). To avoid any surface damage, we did not use conventional Ar-ion thinning but gently crushed the single crystals in ethanol and dispersed them onto an amorphous carbon grid. To remove hydrocarbon contamination, the grid was baked at 160°C for 8 hours in a clean vacuum and then transferred into a microscope. The atomic-resolution ADF STEM images were acquired with an aberration corrected Nion UltraSTEM 200, operated at 200 kV. To avoid beam damage, atomic-resolution observations were performed under a low beam current condition (~9 pA). Under this condition, no significant change can be recognized even scanning more than 30 times over the same region. The image simulation was carried out using the frozen phonon model with a probe-forming aperture half-angle of 30 mrad, the half-angle of the ADF detector spanning 63 to 409 mrad, and incorporating the experimentally-measured, non-uniform detector efficiency^25^.

### XANES experiments

The XANES measurements were carried out using the total electron yield method at BL11A in KEK-PF, Tsukuba, Japan.

### First-principles calculations

The calculations were performed using the projector augmented-wave method^26^ and the HSE06 hybrid functional^20^,^21^ as implemented in the VASP code^27^,^28^. The defects were modeled using 192-atom supercells and spin polarization was allowed for all the defect species and charge states. A plane-wave cutoff energy of 400 eV and the Γ-point only *k*-point sampling were used in the calculations. To correct finite-size effects of the supercells with charged defects, approximate third-order image charge corrections reported in Ref. 29 were applied in conjunction with electrostatic potential alignment suggested in Ref. 30.

## Author Contributions

R.I. and A.R.L. performed electron microscope experiments and prepared the manuscript. F.O. carried out first principles calculations. S.D.F. carried out Z-contrast image simulations. T.T. and K.W. synthesized single crystals and measured photoluminescence spectra. F.O., T.S. and H.H. acquired XANES spectra. N.S., I.T., Y.I. and S.J.P. contributed the discussion and suggestions. All authors read the manuscript.

## Figures and Tables

**Figure 1:**
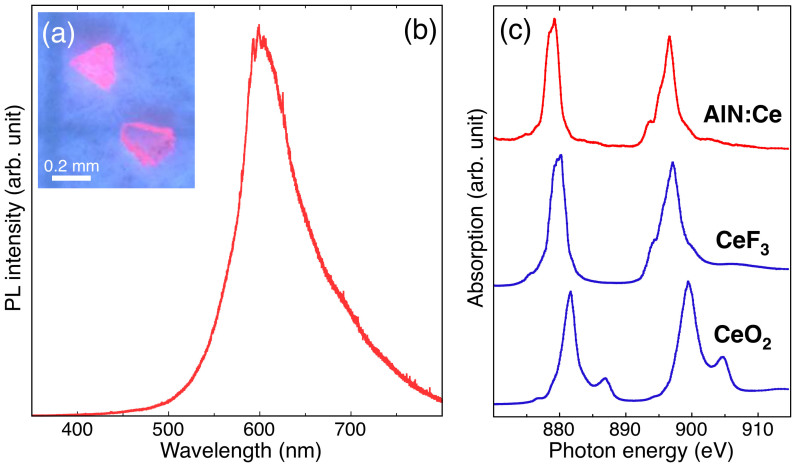
Photoluminescence and XANES spectra obtained from *w*-AlN:Ce single crystals. (a), (b) Pink-colored luminous image obtained by mercury lamp excitation, and the PL spectrum obtained from *w*-AlN:Ce single crystals, where He-Cd laser (325 nm) was used for the excitation source of the PL measurement. (c) Ce *M*_4,5_ XANES spectra obtained from the *w*-AlN:Ce single crystals and reference samples of CeF_3_ (Ce^3+^) and CeO_2_ (Ce^4+^).

**Figure 2:**
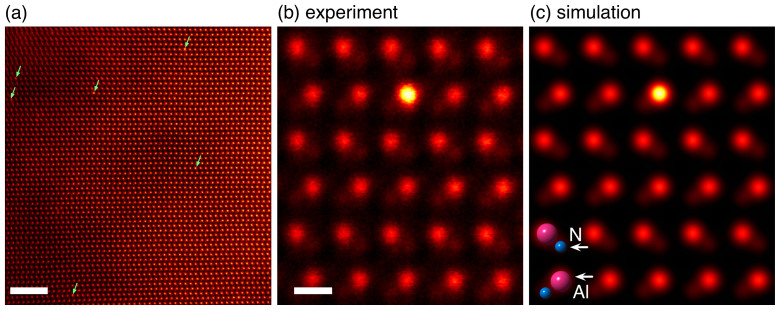
Experimental and simulated Atomic-resolution Z-contrast STEM images. (a) Atomic-resolution ADF STEM image of *w*-AlN:Ce viewed along the 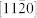 direction. The brighter Al columns marked by arrows contain single Ce atoms. (b) High magnification ADF STEM image, where 10 sequentially acquired frames are averaged after image alignment by cross-correlation; no other image processing is applied. (c) Simulated image based on the neutral Ce_Al_ defect structure with an inserted schematic showing Al and N positions. The specimen thickness is estimated to be 5.3 ± 0.6 nm by quantification of the ADF image^25^ and the Ce atom position is assumed to be at a depth of 1.6 nm. The scale bars are (a) 1 nm and (b) 0.2 nm.

**Figure 3:**
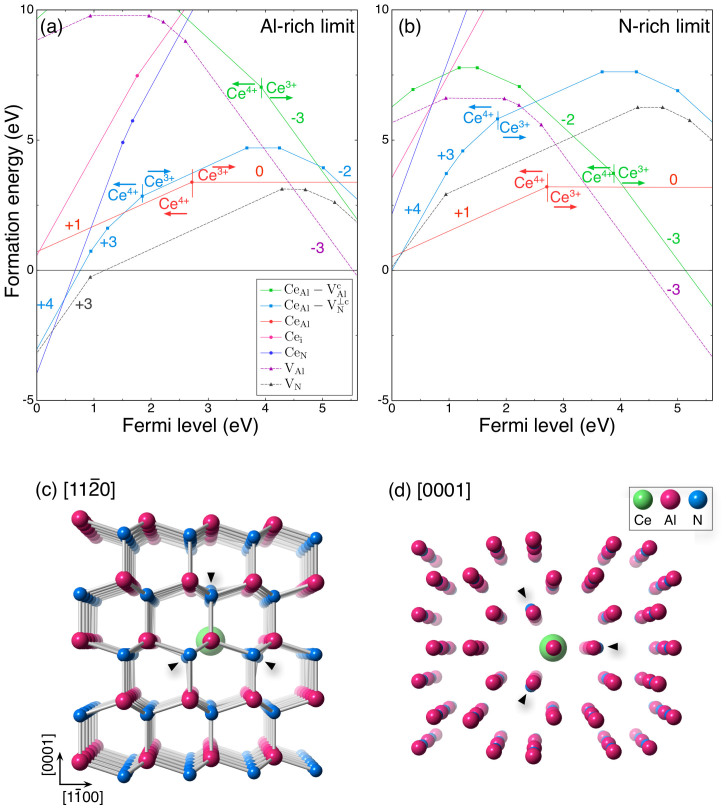
Theoretical formation energies of Ce point-defect structures in *w*-AlN. Formation energies of Ce_Al_*^q^*, Ce_N_*^q^*, Ce_i_*^q^*, (Ce_Al_-V*^i^*_Al_)*^q^*, and (Ce_Al_-V*^j^*_N_)*^q^* defect structures along with V_Al_ and V_N_ as a function of the Fermi level at the chemical potential conditions corresponding to (a) the Al-rich limit and (b) the N-rich limit. The range of the Fermi level is given by the valence band maximum, which is set to be 0 eV, and the conduction band minimum. The slope corresponds to charge state *q*. The formation energies of Ce_Al_-V*^i^*_Al_ (*i* = *a*, *b*, or *c*) and Ce_Al_-V*^j^*_N_ (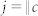 or 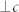) defect complexes show similar behaviors against the Fermi level, respectively, and therefore the most energetically preferable cases of *i* = *c* and *j* = ⊥*c* are plotted in these figures. The relaxed structure of the neutral Ce_Al_ defect viewed along the (c) 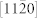 and (d) [0001] directions, where the Ce atom is located at the Al site and the neighboring N atoms show atomic shifts away from the N sites (arrowheads).

## References

[b1] Koizumi S., Watanabe K., Hasegawa M. & Kanda H. Ultraviolet Emission from a Diamond pn Junction. Science 292, 1899–1901 (2001). 11397942 10.1126/science.1060258

[b2] Nakamura S. *et al.* InGaN/GaN/AlGaN-based laser diodes with modulation-doped strained-layer superlattices grown on an epitaxially laterally overgrown GaN substrate. Appl. Phys. Lett. 72, 211–213 (1998).

[b3] Schubert E. F. & Kim J. K. Solid-State Light Sources Getting Smart. Science 308, 1274–1278 (2005). 15919985 10.1126/science.1108712

[b4] Shuji N. *et al.* InGaN-Based Multi-Quantum-Well-Structure Laser Diodes. Jpn J. Appl. Phys. Lett. 35, L74–L76 (1996).

[b5] Taniyasu Y., Kasu M. & Makimoto T. An aluminium nitride light-emitting diode with a wavelength of 210 nanometres. Nature 441, 325–328 (2006). 16710416 10.1038/nature04760

[b6] Hirosaki N. *et al.* Blue-emitting AlN:Eu2+ nitride phosphor for field emission displays. Appl. Phys. Lett. 91, 061101–3 (2007).

[b7] Xie R.-J., Mitomo M., Uheda K., Xu F.-F. & Akimune Y. Preparation and Luminescence Spectra of Calcium- and Rare-Earth (R = Eu, Tb, and Pr)-Codoped alpha-SiAlON Ceramics. J. Am. Ceram. Soc. 85, 1229–1234 (2002).

[b8] Rutz R. F. Ultraviolet electroluminescence in AlN. Appl. Phys. Lett. 28, 379–381 (1976).

[b9] Wolff G. A., Adams I. & Mellichamp J. W. Electroluminescence of AlN. Phys. Rev. 114, 1262–1264 (1959).

[b10] Slack G. A. & McNelly T. F. Growth of high purity AlN crystals. J. Cryst. Growth 34, 263–279 (1976).

[b11] Melnik Y. *et al.* AlN substrates: fabrication via vapor phase growth and characterization. Phys. Stat. Sol. (a) 200, 22–25 (2003).

[b12] Koubaa T. *et al.* Spectra and energy levels of Yb^3+^ in AlN. J. Appl. Phys. 106, 013106–6 (2009).

[b13] Lozykowski H. J., Jadwisienczak W. M. & Brown I. Visible cathodoluminescence of GaN doped with Dy, Er, and Tm. Appl. Phys. Lett. 74, 1129–1131 (1999).

[b14] Lozykowski H. J. & Jadwisienczak W. M. Thermal quenching of luminescence and isovalent trap model for rare-earth-ion-doped AlN. Phys. Stat. Sol. (b) 244, 2109–2126 (2007).

[b15] Vetter U., Zenneck J. & Hofsass H. Intense ultraviolet cathodoluminescence at 318 nm from Gd^3+^-doped AlN. Appl. Phys. Lett. 83, 2145–2147 (2003).

[b16] Taniguchi T. & Yamaoka S. Spontaneous nucleation of cubic boron nitride single crystal by temperature gradient method under high pressure. J. Cryst. Growth 222, 549–557 (2001).

[b17] Taniguchi T. & Watanabe K. Synthesis of high-purity boron nitride single crystals under high pressure by using Ba-BN solvent. J. Cryst. Growth 303, 525–529 (2007).

[b18] Ishikawa R. *et al.* Functional Complex Point-Defect Structure in a Huge-Size-Mismatch System. Phys. Rev. Lett. 110, 065504 (2013). 23432272 10.1103/PhysRevLett.110.065504

[b19] Pennycook S. J. & Boatner L. A. Chemically sensitive structure-imaging with a scanning transmission electron microscope. Nature 336, 565–567 (1988).

[b20] Heyd J., Scuseria G. E. & Ernzerhof M. Hybrid functionals based on a screened Coulomb potential. J. Chem. Phys. 118, 8207–8215 (2003).

[b21] Krukau A. V., Vydrov O. A., Izmaylov A. F. & Scuseria G. E. Influence of the exchange screening parameter on the performance of screened hybrid functionals. J. Chem. Phys. 125, 224106–224105 (2006). 17176133 10.1063/1.2404663

[b22] Hay P. J., Martin R. L., Uddin J. & Scuseria G. E. Theoretical study of CeO_2_ and Ce_2_O_3_ using a screened hybrid density functional. J. Chem. Phys. 125, 034712–8 (2006). 10.1063/1.2206184 16863378

[b23] Da Silva J. L. F., Ganduglia-Pirovano M. V., Sauer J., Bayer V. & Kresse G. Hybrid functionals applied to rare-earth oxides: The example of ceria. Phys. Rev. B 75, 045121 (2007).

[b24] Akamatsu H. *et al.* Strong Spin-Lattice Coupling Through Oxygen Octahedral Rotation in Divalent Europium Perovskites. Adv. Funct. Mater. 23, 1864–1872 (2013).

[b25] Ishikawa R., Lupini A. R., Findlay S. D. & Pennycook S. J. Quantitative Annular Dark Field Electron Microsocpy Using Single Electron Signals. Microsc. Microanal. in press. 10.1017/S1431927613013664 24168987

[b26] Blöchl P. E. Projector augmented-wave method. Phys. Rev. B 50, 17953–17979 (1994). 10.1103/physrevb.50.17953 9976227

[b27] Kresse G. & Furthmüller J. Efficient iterative schemes for ab initio total-energy calculations using a plane-wave basis set. Phys. Rev. B 54, 11169–11186 (1996). 10.1103/physrevb.54.11169 9984901

[b28] Kresse G. & Joubert D. From ultrasoft pseudopotentials to the projector augmented-wave method. Phys. Rev. B 59, 1758–1775 (1999).

[b29] Lany S. & Zunger A. Accurate prediction of defect properties in density functional supercell calculations. Modelling Simul. Mater. Sci. Eng. 17, 084002 (2009).

[b30] Komsa H.-P., Rantala T. T. & Pasquarello A. Finite-size supercell correction schemes for charged defect calculations. Phys. Rev. B 86, 045112 (2012).

